# Future destinations and social inclusion scoping review: how people cured of hepatitis C (HCV) using direct- acting antiviral drugs progress in a new HCV-free world

**DOI:** 10.1186/s13011-022-00475-1

**Published:** 2022-06-08

**Authors:** Sarah R. Donaldson, Andrew Radley, John F. Dillon

**Affiliations:** 1 School of Medicine, University of Dundee, Dundee, DD1 9SY UK; 2 NHS Tayside, Dundee, DD1 9SY UK

**Keywords:** Hepatitis C, Direct-acting antiviral, Identity, Social networks, Recovery capital, Recovery

## Abstract

**Background:**

There has been a paradigm shift in the treatment of Hepatitis C (HCV) from the interferon-era to direct-acting antiviral (DAA) drugs. Cure of HCV for the key risk group, those with a history of injecting drug use, may provide a range of benefits to an individual’s quality of life that can be additional to that of a clinical cure. The interferon-era provided evidence that cure of HCV can be a turning point for those who use drugs, supporting a recovery journey. There remains a question if DAAs can provide the same opportunity.

**Methods:**

We employed a scoping review methodology to consider the additional non-clinical benefits that HCV cure may provide. We used the theoretical construct of recovery capital to consider how these benefits may support a recovery journey in the DAA-era.

**Results:**

Our search provided 2095 articles, from which 35 were included in the analysis. We developed a thematic synthesis of the non-clinical outcomes identified based on the four over-arching themes of recovery capital: physical, cultural, social and human capital. Our review suggests that identity change is a constituent part of each of the recovery capital domains in relation to HCV treatment.

**Conclusion:**

We identified Social Identity Model Of Recovery (SIMOR) as a mechanism through which DAAs may provide non-clinical outcomes to increase recovery capital domains. Further research is required to develop an understanding of the impact a cure of HCV with DAAs may have on identity, overall health and wellbeing and social inclusion to support recovery journeys.

**Supplementary Information:**

The online version contains supplementary material available at 10.1186/s13011-022-00475-1.

## Introduction

Hepatitis C (HCV) is a curable disease. There has been a paradigm shift in HCV treatment from the interferon-era (IFN-era) to the widespread use of direct-acting antiviral (DAA) drugs. DAA drugs can eliminate the virus from the body in a short time with a small treatment burden which is in stark contrast to the experience of the IFN-era [1, 2]. The key group to be treated are those with a history of injecting drug use [3]. Cure of HCV for this key risk group may provide a range of benefits to an individual’s quality of life that may be additional to that of a clinical cure of HCV. These benefits may include improvements in physical and psychological health, social and economic benefits and move away from the stigmatised identity associated with HCV. The IFN-era provided evidence that a cure for HCV can be a turning point for those who use drugs and support a recovery journey. There remains a question if DAAs can provide the same opportunity.

## Background

There is no single consensus of the definition of recovery from substance use; it holds different meanings for different people [4, 5]. In recent years there has been a move away from viewing abstinence as achieving recovery and a shift towards viewing recovery as a process to secure a better life which may still include the use of substances [4]. This journey encompasses other markers of progress towards overall health such as quality of life, wellbeing, building and maintaining relationships and social inclusion (citizenship); living well [6–8]. It is through this lens that we will view the potential of HCV treatment to provide opportunities to support efforts for a “better life”.

The theoretical construct of recovery capital was introduced by Cloud and Granfield [9]. Recovery capital broadly refers to the resources an individual can mobilise to initiate and maintain recovery from dependent substance use: a recovery journey. Recovery capital can be split into four domains which evolve over time and are inter-dependent [10]. Social capital describes the relationships the individual has with others, which may provide physical and emotional support. Cultural capital relates the values, beliefs and attitudes linking the individual to their community and the assets it can provide. Human capital describes positive health and the skills, aspirations and hopes of the individual that are strongly linked to self-efficacy for recovery. Physical capital relates to the tangible assets that an individual has access to such as stable, safe housing, employment or education. Treatment for HCV may support improvements in recovery capital domains and increase overall recovery capital for individuals.

The course of chronic HCV infection is variable. Some people experience few symptoms for long periods of time whilst for others the physical and psychological impact of hepatitis C has a significant influence on daily life and is a strong motivating factor to undergo treatment. The ability to accept treatment can be life-saving [11].

Before 2013, the treatment of chronic HCV infection was based on interferon/ribavirin therapies. The availability of these therapies was limited due to perceptions about patient suitability for treatment by prescribers and payers. Restricted access and the treatment burden associated with these medicines impacted on patient acceptability and compliance and created significant barriers to care. Interferon based regimes are recognised as gruelling and time-consuming, with significant side effects and only a moderate chance of a cure (50–70%) [11–15].

The era of interferon-based regimes required significant stoicism and resilience from the people being treated. Undertaking a course of treatment with these medicines is acknowledged as having a flavour of a personal trial and is described as contributing to a change in personal perspectives and a move beyond substance use; a value greater than cure of a virus [13, 16–19]. This transformation from substance use has been attributed to the toils of interferon and the risk posed by return to a life framed by HCV and another round of treatment to “stay clean”. However, the treatment burden provided by interferon may have resulted in selection bias; in the survival of the fittest – those able to take on the responsibility and demands of therapy engaged with treatment, whilst those unable to do so were left with the burden of disease. For those able to bear the burden of treatment there is uncertainty if transformation potential was already present in these individuals or whether the treatment conferred additional benefit. The fact that they engaged with this arduous therapy may suggest that selected individuals already had the recovery capital required for a journey away from problematic drug use.

The introduction of DAA drugs has transformed HCV care, providing highly effective (95% or greater cure rate), simple and quick treatment regimes with few side effects [20, 21]. The availability of DAAs was expected to improve the treatment potential for the key risk group, those with a history of injecting drug use. Reaching this group who have previously experienced structural barriers such as restricted access to treatment and navigation of complex care systems, make elimination eminently possible [22–25]. However, evidence suggests that significant barriers still exist for some people who inject drugs: concerns about side effects, limited knowledge of HCV, stigma and competing priorities remain in the DAA-era and further action is required to address them [26, 27].

There may be a potential paradox, in that the scale up of HCV treatment with DAAs may mean that those who were previously excluded are now able to access treatment; however, the move from interferon-based treatments to DAA therapies may diminish previously documented turning points for people who use substances. This may be directly due to the less demanding nature of DAA therapy or indirectly through reducing selection bias by lowering treatment barriers and expanding eligibility. The increasing focus on the pill to eliminate the virus may have displaced the delivery of the outcome the patient desires [14].

We employed a scoping review methodology to consider the available research on the additional non-clinical benefits that HCV cure may provide and how these benefits may increase recovery capital, influencing a recovery journey. Our aim is to consider if the potential for transformation from substance use seen in the IFN-era remains with the use of DAAs and consider mechanisms for this change to inform future research.

## Methods

A preliminary search of MEDLINE and the Cochrane Database of Systematic Reviews was conducted and no current or underway systematic or scoping reviews on this topic were identified. We identified that the most suitable type of review to conduct was a scoping review methodology as described by Arksey and O’Malley [28]. The aim of our review was to describe a variety of key factors (non-clinical outcomes of HCV treatment) in relation to the concept of recovery capital. As such we did not aim to answer a specific question, rather provide an overview of the current evidence base, idenfity gaps in current knowledge and inform next steps for research. As scoping reviews do not aim to produce a critical apprasial an assessment of the quality of the evidence is not usually performed [28, 29]. An iterative process was used following the framework set out by Arksey and O’Malley [28] and we were guided by the Preferred Reporting Items for Systematic reviews and Meta-Analyses extension for Scoping Reviews (PRISMA-ScR) Checklist [30]. A copy of the PRISMA-ScR Checklist is reported in Additional file 1.

### Identifying the broad research question

- To explore the non-clinical themes of a cure of HCV for people with a history of injecting drug use in relation to a recovery journey.
- Survey the current literature for evidence of DAAs providing these benefits and potential mechanisms for this change to inform future research.

### Eligibility criteria

#### Participants

Literature relating to people with hepatitis C and a history of injecting drug use.

#### Concept

Literature describing non-clinical outcomes as a result of hepatitis C treatment.

#### Context

Literature in English from 1991 onwards, as this is the year that interferon became commercially available to treat chronic HCV infection. This scoping review will consider both qualitative and quantitative literature. In addition systematic reviews, book chapters and editorials that meet the review criteria are included in the scope.

### Identifying relevant studies

The initial search was conducted in November 2019 from five databases: Ovid Medline, Ovid Embase, Web of Science (core collection), Sociological Abstracts and CINAHL. A grey literature search was conducted using Google Scholar. Search terms were customised for each database. Reference lists of articles identified as suitable were hand screened for any additional relevant articles. Initial searches showed that non-clinical benefits evidenced in the interferon-era related to recovery from substance use and transformation of quality of life, wellbeing and a move beyond substance use. The search strategy was refined to include terms relating to these non-clinical outcomes. The search strategy was repeated in December 2021 to capture any recently published evidence. Search terms are shown in Additional file 2.

### Screening

Following the search all articles identified were transferred to EndNote X9 and duplicates removed. Titles and abstracts were screened by two authors (AR and SRD) for assessment against the eligibility criteria. Articles identified as potentially relevant were then subject to full article screening independently by two authors (AR and SRD) where there was disagreement this was resolved through discussion and consensus reached. A third author (JFD) was available for consultation; however this was not necessary during this process.

### Selection of studies

**Fig. 1:**
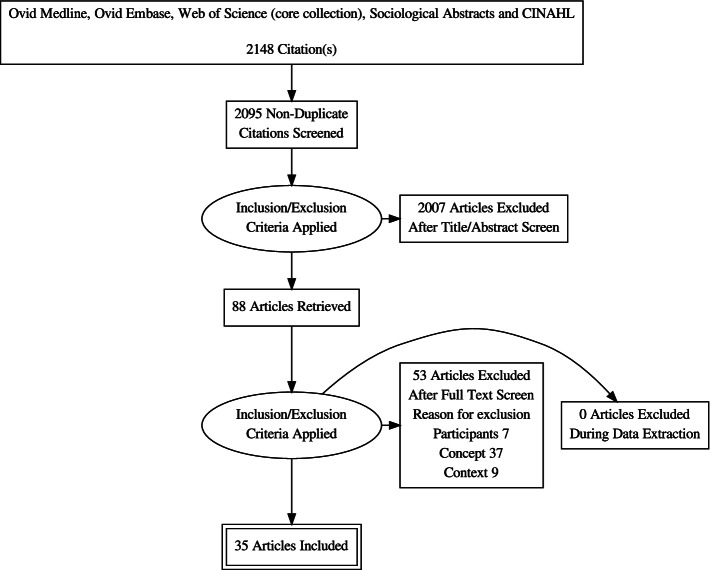
PRISMA flow diagram of study selection

Two thousand one hundred forty-eight items were title screened from the database searches, 14 from grey literature and 11 from hand searching. Duplicate results were excluded resulting in 2095 articles for title and abstract screening. After title and abstract screening 88 articles remained for full article screening. The full article review yielded 35 papers for data extraction. No articles were excluded during data extraction as this is a scoping review and therefore a quality assessment was not conducted. A PRISMA diagram of study selection is shown in Fig. 1 below [30, 31].

### Charting the data

Data relating to each article including geographical location, methods and key findings were extracted and recorded in an excel spreadsheet through consensus of two authors (AR and SRD). The data extracted is shown in Additional file 3. In categorising the literature there is a risk that generalisations are made, however the approach provides a framework for thematic analysis which is described below.

## Results

The general scope and distribution of the literature included in this scoping review is presented in Table 1.

**Table 1:** Scope and distribution of literature

Publication type	Number (***n*** = 35)
Non-original research (*n* = 8)	
Review papers	5
Reports/books chapters/editorials	3
Original research (*n* = 27)	
Qualitative	22
Quantitative	3
Mixed-methods	2
*Origin of original research (n = 27)*	
*Australia*	*8*
*Europe*	*2*
*New Zealand*	*2 (1 multi-site with Australia)*
*UK*	*7*
*USA*	*5*
*Canada*	*3*

### Descriptive analysis of selected articles

We used an excel sheet to code each article by intervention and non-clinical outcome(s) and recovery capital domain. Two authors (AR and SRD) reviewed each article and a consensus reached as to relevant coding.

### Narrative synthesis

We clustered the non-clinical outcomes identified in the descriptive analysis of the selected articles by recovery capital domain to explore potential mechanisms for DAAs to support a recovery journey. We developed a thematic synthesis based on the four over arching themes of recovery capital: physical, cultural, social and human capital.

### Cultural capital

“Hep C’s like the common cold for the junkie” [32].

HCV may act as a symbolic marker of injecting drug use, providing an identity of “junkie”, “dirty”, “risky” and beyond the control of the individual. HCV is framed as a shameful and stigmatising disease but also one of passivity and helplessness [33–35]. People who inject drugs break the norms of a society in which drug use is perceived as wrong; as a result of a flawed character resulting in exclusion from wider society; the spoiled identity and the second class citizen [34]. Experiences of stigma and discrimination are common and clearly documented for people in both the interferon [19, 36–38] and DAA era [39]; this creates strong barriers to accessing HCV care and treatment.

Harris [40] and Dowsett et al. [33] describe a range of reactions to a HCV diagnosis from devastating for some to little concern for others. There is a clear narrative in the interferon-era documenting a culture of inevitability of HCV in this patient group. HCV is described as being largely socially accepted and expected within injecting drug using social networks; HCV is a burden to bear and this may reinforce a stigmatised identity [26, 33, 37, 38, 40]. This narrative has not changed with advances in treatment [32, 41].

Competing priorities may be the biggest challenge to overcome this perceived inevitability. This is especially relevant for those who are asymptomatic and may provide an explanation for low engagement by some despite the availability of DAAs [32, 41–44].

Awareness of HCV status was found to reduce quality of life due to the psychological impact of receiving a positive diagnosis and the effect of labelling with a stigmatised disease [45]. The systematic review by Dowsett et al. [33] describes the experiences of living with HCV and the emotional responses to the stigma experienced to include shame, feelings of being “dirty” and rejection, resulting in reduced quality of life. These responses were also described in Harris’s [35] qualitative study and demonstrate the lack of movement in social acceptance of those who use drugs. This is reinforced by Whiteley et al. [46] who describe the “cultural lag” of DAAs ability to influence the stigma surrounding HCV diagnosis and treatment which is still framed in the interferon-era.

Rhodes et al. [19] described the transformative potential in the interferon-era and the paradox of therapeutic citizenship dividing those who could present as responsible patients suitable for treatment and those who continue to use drugs as beyond hope. Restrictions on access to HCV treatment have reinforced pervasive social narratives about worthiness of those who use drugs for treatment for hepatitis C, with patients reporting gratitude rather than expectation of treatment [19, 42]. This narrative has evolved with treatment options from perceived inability to comply in the interferon-era to questions of cost of treatment and merit to receive scarce resources in the DAA era [32, 39, 41, 44, 46].

Rance et al. [47] describe the politics surrounding pharmaceutical citizenship in the DAA-era, where patient friendly DAAs are heralded as important for the HCV community to open up access and increase inclusion. The transformation in HCV care has been enthusiastically embraced by clinicians, however, has yet to be widely accepted by patients due to a culture of caution, confusion and doubt [27, 39]. At a patient level there are descriptions of disappointment and scepticism that a pill could deliver the hoped for social transformation against a back drop of complex and long standing social problems and stigma [41, 47].

The legacy of the interferon-era continues to shape how people who use drugs perceive HCV, their expectation and experience of treatment [39, 41]. The interferon-era was synonymous with debilitating side effects and as a result patients are described to have anticipation of a similar experience with DAAs [27, 41, 46]. Whilst the expectation deters some from treatment the lack of side effects from DAAs is almost seen as problematic for others, who describe side effects as practically desirable, as evidence that treatment is working and “no pain, no gain” [46]: A way of earning a cure. This reinforces the culture that patients must be deserving of treatment.

Cultural barriers remain for people with HCV despite the paradigm shift in care. Cure of HCV with DAAs does however, have the potential to increase cultural recovery capital. As HCV treatment becomes more inclusive, with fewer barriers and restrictions for use this may begin to address some of the beliefs of inevitability and address some of the stigma surrounding HCV.

### Social capital

For some, the stigma associated with HCV is a motivating factor for undergoing treatment. Treatment may provide the opportunity to move away from a stigmatised identity and to restore social membership. This may be reinforced by the opportunity to strengthen relationships with partners and family. This was demonstrated in both the interferon-era [13, 48] and the DAA-era [48–52]. Doing so however, comes at a personal cost with individuals reporting negative experiences from their interactions with the complex healthcare system in both the interferon-era [27, 33, 38, 40] and DAA-era [27, 33, 41, 44].

Goodyear et al. [52] found that for some, treatment with DAAs fell short of the desired social transformation hoped for. This is attributed to the remaining socio-structural challenges and stigma faced such as those as a result of substance use, poverty and other health conditions such as HIV. However, for others the reduction of internalised stigma and the opportunity for new identities and social connections supported a recovery journey.

Falade-Nwulia et al. [1] describe the influence of the peer network related to health behaviours of the individual in the DAA-era through knowledge exchange and the influence of the social norms. Social networks can facilitate HCV treatment or be a barrier to engagement. Whilst those treated with DAAs are keen to share positive experiences and encourage others to engage there is trepidation to share this information due to the return to a past stigmatised identity [49].

Cure of HCV is described as an opportunity for a new identity, one of being “clean” and a break from their past using identity across both treatment regimes [35, 49]. For those undergoing treatment with DAAs, social redemption is described as a key motivating factor [51]. When HCV cure is achieved, this is suggested to translate to a change in self-identity resulting in the formation of new social networks away from injecting associations, supporting a recovery journey in the IFN-era [38]. Treatment with DAAs is suggested to reinforce the pursuit of a recovery journey with individuals taking steps to reduce the risk of re-infection [51–54].

Rance et al. [18] describe the benefits of introducing HCV care into opiate substitution therapy (OST) clinics during the interferon-era to provide a holistic form of care. The study found that this shifted the dynamics of the relationship between staff and patients, and this therapeutic relationship opened up opportunities for transformation and a shift in identity towards that of “non-addicts” from the stigmatised “drug user identity”. The clinician-patient relationship required in the interferon-era provided broader care beyond the virus and there are concerns that the reduced relationship in the DAA-era may be detrimental to these social supports [14]. Goodyear et al. [41] identified further barriers to care in the DAA era based around perceived gate keeping and lack of information from care providers. These concerns may be addressed through comprehensive health and social services in the DAA-era [41, 44, 51].

Concerns about HCV transmission is identified as a motivating factor to undergo treatment in order to not pass it on to others across the paradigm shift [33, 49, 55]. Richmond et al. [49] describes the reduced psychological burden for individuals cured with DAAs through feeling “normal” and not an infectious risk to others. This may encourage the re-establishment of closer relationships or provide confidence to build new ones.

HCV cure may increase social recovery capital through the strengthening of relationships, the building of new non-injecting social networks and the removal of the risk of HCV transmission to others.

### Human capital

A focus on the priorities and wishes of people who use drugs from HCV treatment could hold the answer to widening access and overcoming remaining barriers. Madden et al. [56] propose that in the DAA-era people who inject drugs may be looking for a cure beyond HCV; the opportunity for social redemption and an untainted identity away from the stigmatising disease. This view is shared by Harris [48], Williams et al. [51] and Bryant et al. [27] who describe the promise of DAA treatment and its potential of new opportunities of a new identity or “normality”.

Richmond et al. [49] describe the psychological relief for those cured of HCV with DAAs, even if they did not experience physical symptoms. The removal of the uncertainty of HCV infection and its potential to impact on future health is seen as a positive benefit, with hope for the future.

Treatment with DAAs has been shown to improve physical health and wellbeing which support feelings of pride and achievement of obtaining a cure and hope for a better life and future aspirations [52].

Non-clinical outcomes such as improved wellbeing, increase in self-esteem and the ability to plan and look to the future are beginning to emerge as a result of treatment with DAAs [39, 51, 57].

Cure of HCV may increase human recovery capital. The opportunity to increase hope and aspirations for the future and the potential for a new identity or recovery of an old valued identity.

### Physical capital

The psychological, physical and social aspects of living with HCV burden are widely documented [33, 38]. HCV can impact on daily life, quality of life and access to sources of physical capital due to the symptoms of the disease [33, 52]. People living with chronic HCV experience a range of physical symptoms such as fatigue, weakness, nausea, pain, headaches and psychological symptoms such as depression, anxiety and irritability [33, 55, 58, 59]. The physical and mental fatigue arising from these symptoms has been found to frame a person’s social interactions and activities and disrupt daily life [58, 59]. The resulting changes to employment status and social roles have been described as having implications for finances and morale.

The improvements in physical health as a result of successful DAA treatment provides support in obtaining and maintaining employment [52]. Building opportunities for social activities, education and employment may provide opportunity for a new identity or reclaiming old valued identities lost as a result of changes to employment and social roles.

Cure of HCV provides an opportunity to increase physical recovery capital by easing physical and psychological symptoms experienced as a result of infection. This may increase opportunities for social activities, employment and education.

## Discussion

The aim of this scoping review was to explore themes in current knowledge relating to non-clinical outcomes of HCV treatment, going beyond the “personal trial” of the interferon-era and considering how HCV treatment may influence a recovery journey. Our review suggests that HCV cure has the potential to increase recovery capital through the four domains; physical, cultural, social and human. This increase in recovery capital may provide an opportunity for the initiation and maintenance of a recovery journey.

HCV treatment may increase opportunities for social activities, education and employment by addressing physical and psychological symptoms of HCV which impact on daily life [33, 38, 52, 55, 58, 59]. The culture of a stigmatised identity linked to substance use; the inevitability of disease reinforcing this identity and pervasive narratives of worthiness of treatment may be addressed by inclusive HCV treatment [32, 39, 41, 44, 46]. As described by a number of studies HCV treatment may provide a catalyst for some to alter social relationships through a shift in social networks away from injecting networks and towards networks supportive of a recovery journey [1, 13, 48–52]. Our scoping review found narratives of patient desires for a positive future new identity or the reclaiming an old valued identity which provides the prospect of hope and fulfilled aspirations [27, 39, 48, 49, 51, 52, 56, 57].

The findings from our scoping review suggest that identity change is a constituent part of each of the recovery capital domains in relation to HCV treatment. Identity transformation is recognised as a key component of recovery from substance use [60–66]. People socialise with others who hold similar views, attitudes, values and life styles; where certain behaviours are the norm [67]. Our self-identity is derived from people in our social network and this often shapes our behaviour, influencing health and wellbeing [63, 66–69]. Social identity can provide support in stressful situations and guide positive lifestyle choices. Social identity may also influence negative lifestyle choices with a group’s behavioural norm creating a barrier to recovery [69, 70]. Social networks have been found to have the potential to positively or negatively impact on HCV treatment uptake [1].

The social identity model of recovery (SIMOR) suggests that recovery from substance use relies on a change in self-identity, which in turn shapes the social network of the individual towards actively engaging with a network where substance use is not the norm [64, 71–73]. This social network, where substance use is not the norm provides support for recovery and a new focus on tangible assets such as relationships, education, volunteering and work [66]. The entwined nature of individual and social identity means that they are likely to reinforce each other. The differing behaviours and norms required in order to be accepted in a new network are difficult to obtain without a different identity. Whilst the importance of social identity has previously been described for people who use drugs to enter recovery, it remains unclear as to effective mechanism(s) to spark this change in self –identity.

Recognising and harnessing the potential of DAAs to influence identity may enable us to realise their health-enhancing potential wider than a clinical cure and deliver a potential cure that patients desire [27, 48, 51, 52, 56, 66]. Developing the understanding of how cure of HCV may influence identity, recovery journeys and social inclusion may therefore provide compelling evidence for embracing DAAs potential, overcoming a legacy of interferon and address the wider health needs for people who use drugs [50]. Future research should focus on the types of treatment or interventions that provide a mechanism for a shift in self and social identity which is supportive of a recovery journey to consider evidence for the hypothesis that cure of HCV with DAAs may provide this opportunity.

### Limitations

The purpose of a scoping review is to provide an overview of a particular topic and therefore includes multiple types of work. As such an assessment of the quality of the work is not conducted and therefore there are limitations in the applicability to practice, where concrete guidance may be required. We did not register the protocol for our scoping review which limits transparency and replicability. Our work only identified 35 studies over a 40 year period from countries with a western culture (Australia, New Zealand, Europe and the USA). The differing health systems, policies and treatment practices may impact significantly on the findings of the review. Future work should consider broadening the search criterion to include other health conditions, such as HIV, which may provide valuable transferable knowledge and understanding. Our review also suggests that there is a weighting towards some recovery capital domains over others, the weighting of the non-clinical outcomes is towards social and cultural domains which are external resources that may not be under the influence of the individual. These limitations combined may therefore affect the generalisability of the conclusions drawn.

## Conclusion

Treatment with DAAs has the potential to engage many more people from marginalised populations, curing the infection and moving to elimination at a population level. However, currently HCV treatment is framed with the context of eliminating the virus when it actually needs to be framed in the context of patient’s desires from treatment; social redemption and a move away from a stigmatised identity. The structural changes we need to deliver the elimination of HCV are likely to include recognition and delivery of non-clinical benefits: a challenge for the way we conceptualise and design pathways of care. This scoping review identified that HCV treatment may increase the recovery capital available to the individual to be brought to bear on the initiation and maintenance of a recovery journey. HCV treatment may influence each of the four suggested domains of recovery capital; physical, cultural, social and human capital through the mechanism of the social identity model of recovery.

Further research is required to develop an understanding of the impact a cure of HCV with DAAs may have on identity and overall health and wellbeing of the individual and their social networks. This may provide an opportunity to shift the perception of HCV treatment from the legacy of interferon to a wider view of improving patient experiences of wellbeing, inclusion and supporting recovery journeys; patient’s desires and hopes of treatment. Developing this knowledge of the broader benefits that DAAs may provide may increase patient and payers enthusiasm for embracing the potential of DAAs.

## Supplementary Information

**Additional file 1.** Preferred Reporting Items for Systematic reviews and Meta-Analyses extension for Scoping Reviews (PRISMA-ScR) Checklist.

**Additional file 2.** Search Terms.

**Additional file 3.** Description of articles and themes.

## Data Availability

The datasets used and/or analysed during the current study are available from the corresponding author on reasonable request.

## Abbreviations

DAA: Direct-Acting Antiviral
HCV: Hepatitis C
IFN: Interferon
SIMOR: Social Identity Model of Recovery
PRISMA-ScR: Preferred Reporting Items for Systematic reviews and Meta-Analyses extension for Scoping Reviews

## References

[1] Falade-Nwulia O, Sacamano P, McCormick SD, Yang C, Kirk G, Thomas D, Sulkowski M, Latkin C, Mehta SH (2020). Individual and network factors associated with HCV treatment uptake among people who inject drugs. Int J Drug Policy.

[2] WHO. Guidelines for the screening care and treatment of persons with chronic hepatitis C infection: World health Organization; 2016. 27227200

[3] European monitoring Centre for Drugs and Drug Addiction (EMCDDA) (2016). Hepatitis C among drug users in Europe: epidemiology, Treatment and prevention.

[4] Ashford RD, Brown A, Brown T, Callis J, Cleveland HH, Eisenhart E, Groover H, Hayes N, Johnston T, Kimball T, Manteuffel B (2019). Defining and operationalizing the phenomena of recovery: a working definition from the recovery science research collaborative. Addict Res Theory.

[5] The Betty Ford Institute Consensus Panel (2007). What is recovery? A working definition from the Betty ford institute. J Subst Abus Treat.

[6] White WL (2007). Addiction recovery: its definition and conceptual boundaries. J Subst Abus Treat.

[7] Best D, Laudet A (2010). The potential of recovery capital.

[8] Cano I, Best D, Edwards M, Lehman J (2017). Recovery capital pathways: Modelling the components of recovery wellbeing. Drug Alcohol Depend.

[9] Cloud W, Granfield R (2008). Conceptualizing recovery capital: expansion of a theoretical construct. Subst Use Misuse.

[10] Hennessy EA (2017). Recovery capital: a systematic review of the literature. Addict Res Theory.

[11] WHO. Combating hepatitis B and C to reach elimination by 2030: advocacy brief: World health Organization; 2016.

[12] Bonaccorso S, Marino V, Biondi M, Grimaldi F, Ippoliti F, Maes M (2002). Depression induced by treatment with interferon-alpha in patients affected by hepatitis C virus. J Affect Disord.

[13] Clark JA, Gifford AL (2014). Resolute efforts to cure hepatitis C: understanding patients’ reasons for completing antiviral treatment. Health.

[14] Harris M, Rhodes T (2018). Caring and curing: considering the effects of hepatitis C pharmaceuticalisation in relation to non-clinical treatment outcomes. Int J Drug Policy.

[15] Körner H (2010). Negotiating treatment for hepatitis C: interpersonal alignment in the clinical encounter. Health.

[16] Jones L, Atkinson A, Bates G, McCoy E, Porcellato L, Beynon C, McVeigh J, Bellis MA (2014). Views and experiences of hepatitis C testing and diagnosis among people who inject drugs: systematic review of qualitative research. Int J Drug Policy.

[17] Newman AI, Beckstead S, Beking D, Finch S, Knorr T, Lynch C, MacKenzie M, Mayer D, Melles B, Shore R (2013). Treatment of chronic hepatitis C infection among current and former injection drug users within a multidisciplinary treatment model at a community health Centre. Can J Gastroenterol.

[18] Rance J, Treloar C, ETHOS Study Group (2014). ‘Not just M ethadone T racy’: transformations in service-user identity following the introduction of hepatitis C treatment into a ustralian opiate substitution settings. Addiction..

[19] Rhodes T, Harris M, Martin A (2013). Negotiating access to medical treatment and the making of patient citizenship: the case of hepatitis C treatment. Sociol Health Illness.

[20] Falade-Nwulia O, Suarez-Cuervo C, Nelson DR, Fried MW, Segal JB, Sulkowski MS (2017). Oral direct-acting agent therapy for hepatitis C virus infection: a systematic review. Ann Intern Med.

[21] Pawlotsky JM, Negro F, Aghemo A, Berenguer M, Dalgard O, Dusheiko G, Marra F, Puoti M, Wedemeyer H (2020). European Association for the Study of the liver. EASL recommendations on treatment of hepatitis C: final update of the series☆. J Hepatol.

[22] Bruggmann P, Grebely J (2015). Prevention, treatment and care of hepatitis C virus infection among people who inject drugs. Int J Drug Policy.

[23] Lazarus JV, Wiktor S, Colombo M, Thursz M (2017). Micro-elimination–a path to global elimination of hepatitis C. J Hepatol.

[24] Radley A, Robinson E, Aspinall EJ, Angus K, Tan L, Dillon JF (2019). A systematic review and meta-analysis of community and primary-care-based hepatitis C testing and treatment services that employ direct acting antiviral drug treatments. BMC Health Serv Res.

[25] Wade AJ, Doyle JS, Gane E, Stedman C, Draper B, Iser D, Roberts SK, Kemp W, Petrie D, Scott N, Higgs P (2020). Outcomes of treatment for hepatitis C in primary care, compared to hospital-based care: a randomized, controlled trial in people who inject drugs. Clin Infect Dis.

[26] Davis M, Rhodes T (2004). Beyond prevention? Injecting drug user narratives about hepatitis C. Int J Drug Policy.

[27] Bryant J, Rance J, Hull P, Mao L, Treloar C (2019). Making sense of ‘side effects’: Counterpublic health in the era of direct-acting antivirals. Int J Drug Policy.

[28] Arksey H, O'Malley L (2005). Scoping studies: towards a methodological framework. Int J Soc Res Methodol.

[29] Peters MD, Godfrey CM, Khalil H, McInerney P, Parker D, Soares CB (2015). Guidance for conducting systematic scoping reviews. Int J Evid Based Healthcare.

[30] Tricco AC, Lillie E, Zarin W, O'Brien KK, Colquhoun H, Levac D, Moher D, Peters MD, Horsley T, Weeks L, Hempel S (2018). PRISMA extension for scoping reviews (PRISMA-ScR): checklist and explanation. Ann Intern Med.

[31] Peters MD, Marnie C, Tricco AC, Pollock D, Munn Z, Alexander L, McInerney P, Godfrey CM, Khalil H (2020). Updated methodological guidance for the conduct of scoping reviews. JBI Evid Synthesis.

[32] Skeer MR, Ladin K, Wilkins LE, Landy DM, Stopka TJ (2018). 'Hep C's like the common cold': understanding barriers along the HCV care continuum among young people who inject drugs. Drug Alcohol Depend.

[33] Dowsett LE, Coward S, Lorenzetti DL, MacKean G, Clement F (2017). Living with hepatitis C virus: a systematic review and narrative synthesis of qualitative literature. Can J Gastroenterol Hepatol.

[34] Treloar C, Rance J, Backmund M (2013). Understanding barriers to hepatitis C virus care and stigmatization from a social perspective. Clin Infect Dis.

[35] Harris M (2009). Injecting, infection, illness: abjection and hepatitis C stigma. Body Soc.

[36] Fraser S, Treloar C (2006). 'Spoiled identity' in hepatitis C infection: the binary logic of despair. Crit Public Health.

[37] Rhodes T, Treloar C (2008). The social production of hepatitis C risk among injecting drug users: a qualitative synthesis. Addiction..

[38] Treloar C, Rhodes T (2009). The lived experience of hepatitis C and its treatment among injecting drug users: qualitative synthesis. Qual Health Res.

[39] Whiteley D, Whittaker A, Elliott L, Cunningham-Burley S (2018). Hepatitis C in a new therapeutic era: Recontextualising the lived experience. J Clin Nurs.

[40] Harris M (2009). Troubling biographical disruption: narratives of unconcern about hepatitis C diagnosis. Sociol Health Illness.

[41] Goodyear T, Brown H, Browne AJ, Hoong P, Ti L, Knight R (2021). “I want to get better, but…”: identifying the perceptions and experiences of people who inject drugs with respect to evolving hepatitis C virus treatments. Int J Equity Health.

[42] Giraudon I, Wiessing L, Hedrich D, Kalamara E, Griffiths P, Simon R (2016). Hepatitis C among drug users in Europe: epidemiology, Treatment and prevention: Publication Office of the European Union.

[43] Razavi H, Sanchez Gonzalez Y, Yuen C, Cornberg M. Global timing of hepatitis C virus elimination in high‐income countries. Liver International. 2020 Mar;40(3):522–9. 10.1111/liv.14324 31815353

[44] Goodyear T, Ti L, Carrieri P, Small W, Knight R (2020). “Everybody living with a chronic disease is entitled to be cured”: challenges and opportunities in scaling up access to direct-acting antiviral hepatitis C virus treatment among people who inject drugs. Int J Drug Policy.

[45] McDonald SA, Hutchinson SJ, Palmateer NE, Allen E, Cameron SO, Goldberg DJ, Taylor A (2013). Decrease in health-related quality of life associated with awareness of hepatitis C virus infection among people who inject drugs in Scotland. J Hepatol.

[46] Whiteley D, Whittaker A, Elliott L, Cunningham-Burley S (2016). The lived experience of interferon-free treatments for hepatitis C: a thematic analysis. Int J Drug Policy.

[47] Rance J, Rhodes T, Lancaster K. Pharmaceutical citizenship in an era of universal access to hepatitis C treatment: situated potentials and limits. Health. 2021. 10.1177/1363459320988887. 10.1177/1363459320988887 33506718

[48] Harris M (2017). Managing expense and expectation in a treatment revolution: problematizing prioritisation through an exploration of hepatitis C treatment ‘benefit’. Int J Drug Policy.

[49] Richmond JA, Ellard J, Wallace J, Thorpe R, Higgs P, Hellard M, Thompson A (2018). Achieving a hepatitis C cure: a qualitative exploration of the experiences and meanings of achieving a hepatitis C cure using the direct acting antivirals in Australia. Hepatol Med Policy.

[50] Grebely J, Hajarizadeh B, Lazarus JV, Bruneau J, Treloar C (2019). International network on hepatitis in substance users. Elimination of hepatitis C virus infection among people who use drugs: ensuring equitable access to prevention, treatment, and care for all. Int J Drug Policy.

[51] Williams BE, Nelons D, Seaman A, Witkowska M, Ronan W, Wheelock H, Zaman A, Garcia J (2019). Life projects: the transformative potential of direct-acting antiviral treatment for hepatitis C among people who inject drugs. Int J Drug Policy.

[52] Goodyear T, Brown H, Browne AJ, Hoong P, Ti L, Knight R. “Stigma is where the harm comes from”: Exploring expectations and lived experiences of hepatitis C virus post-treatment trajectories among people who inject drugs. Int J Drug Policy. 2021;96:103238. 10.1016/j.drugpo.2021.103238 PMC8881088 33902968

[53] Caven M, Malaguti A, Robinson E, Fletcher E, Dillon JF (2019). Impact of hepatitis C treatment on behavioural change in relation to drug use in people who inject drugs: a systematic review. Int J Drug Policy.

[54] Pourmarzi D, Smirnov A, Hall L, FitzGerald G, Rahman T (2020). ‘I’m over the moon!’: Patient-perceived outcomes of hepatitis C treatment. Aust J Primary Health.

[55] Conrad S, Garrett L, Cooksley W, Dunne M, MacDonald G (2006). Living with chronic hepatitis C meansyou just haven't got a normal life any more'. Chronic Illness.

[56] Madden A, Hopwood M, Neale J, Treloar C. Beyond cure: patient reported outcomes of hepatitis C treatment among people who inject drugs in Australia. Harm Reduction Journal. 2018;15(1):1–8. 10.1186/s12954-018-0248-4 PMC6094926 30111327

[57] Torrens M, Soyemi T, Bowman D, Schatz E (2020). Beyond clinical outcomes: the social and healthcare system implications of hepatitis C treatment. BMC Infect Dis.

[58] Dunne EA, Quayle E (2001). The impact of iatrogenically acquired hepatitis C infection on the well-being and relationships of a group of Irish women. J Health Psychol.

[59] Groessl EJ, Weingart KR, Kaplan RM, Clark JA, Gifford AL, Ho SB (2008). Living with hepatitis C: qualitative interviews with hepatitis C-infected veterans. J Gen Intern Med.

[60] Dingle GA, Cruwys T, Frings D (2015). Social identities as pathways into and out of addiction. Front Psychol.

[61] Frings D, Albery IP (2015). The social identity model of cessation maintenance: formulation and initial evidence. Addict Behav.

[62] Praharso NF, Tear MJ, Cruwys T (2017). Stressful life transitions and wellbeing: a comparison of the stress buffering hypothesis and the social identity model of identity change. Psychiatry Res.

[63] Mawson E, Best D, Beckwith M, Dingle G, Lubman D (2015). Social identity, social networks and recovery capital in emerging adulthood: a pilot study. Subst Abuse Treat Prev Policy.

[64] Best D, Beckwith M, Haslam C, Alexander Haslam S, Jetten J, Mawson E, Lubman DI (2016). Overcoming alcohol and other drug addiction as a process of social identity transition: the social identity model of recovery (SIMOR). Addict Res Theory.

[65] McIntosh J, McKeganey N (2000). Addicts' narratives of recovery from drug use: constructing a non-addict identity. Soc Sci Med.

[66] Jetten J, Haslam C, Alexander SH. The social cure: identity, health and well-being: Psychology press; 2012.

[67] Giovazolias T, Themeli O (2014). Social learning conceptualization for substance abuse: Implications for therapeutic interventions.

[68] West R (2013). European monitoring Centre of Drugs and Drug Addiction (EMCDDA), Insights. Models of Addiction. EMCDDA.

[69] Smith KP, Christakis NA (2008). Social networks and health. Annu Rev Sociol.

[70] Johnstone M, Jetten J, Dingle GA, Parsell C, Walter ZC (2015). Discrimination and well-being amongst the homeless: the role of multiple group membership. Front Psychol.

[71] Bathish R, Best D, Savic M, Beckwith M, Mackenzie J, Lubman DI (2017). “Is it me or should my friends take the credit?” the role of social networks and social identity in recovery from addiction. J Appl Soc Psychol.

[72] Dingle GA, Stark C, Cruwys T, Best D (2015). Breaking good: breaking ties with social groups may be good for recovery from substance misuse. Br J Soc Psychol.

[73] Collinson B, Best D (2019). Promoting recovery from substance misuse through engagement with community assets: asset based community engagement. Subst Abus.

