# Supplementary material for: Future destinations and social inclusion scoping review: how people cured of hepatitis C (HCV) using direct- acting antiviral drugs progress in a new HCV-free world
Source: Subst Abuse Treat Prev Policy. 2022 Jun 8;17:45. doi: 10.1186/s13011-022-00475-1 (PMC9178822; doi:10.1186/s13011-022-00475-1)
Supplement: Supplementary file 2 — Additional file 2. Search Terms. [file 13011_2022_475_MOESM2_ESM.docx]

# Additional file 2

Search Terms

1: "hepatitis C" or "HCV" or “HCV treatment” or “hepatitis C treatment” or “Interferon” or “Direct-Acting Antivirals” or “DAA”

2: "Substance abuse" or "substance misuse" or "substance use" or "substance dependence" or "Drug abuse" or "drug misuse" or "drug use" or "drug dependence" or "injecting drug use"

3: "recovery" or "recovery capital" or "recovery journey" or "citizenship" or "social inclusion" or "social exclusion" or "identity" or "self-identity" or "social network" or "wellbeing" or "quality of life" or "QoL"

Searches 1+2, 1+2+3,
