# Supplementary material for: Future destinations and social inclusion scoping review: how people cured of hepatitis C (HCV) using direct- acting antiviral drugs progress in a new HCV-free world
Source: Subst Abuse Treat Prev Policy. 2022 Jun 8;17:45. doi: 10.1186/s13011-022-00475-1 (PMC9178822; doi:10.1186/s13011-022-00475-1)
Supplement: Supplementary file 3 — Additional file 3. Description of articles and themes. [file 13011_2022_475_MOESM3_ESM.docx]

# Additional file 3

# Description of articles and themes

| **Author (Year)** | **Journal/Book** | **Country** | **Method** | **Intervention** | **Theme** | **Recovery Capital Domain** |
| --- | --- | --- | --- | --- | --- | --- |
| Bryant et al. (2019) | International Journal of Drug Policy | Australia | Mixed methods | DAA | Side effect of DAAs and narrative surrounding side effects | Cultural, Social, Human |
| Caven et al. (2019) | International Journal of Drug Policy |  | Systematic Review | IFN & DAA | Changes in drug use when undergoing HCV treatment | Social |
| Clark and Gifford (2014) | Health | USA | Qualitative ethnography | IFN | Patient reasons for completing HCV treatment | Social |
| Conrad et al. (2006) | Chronic Illness | Australia | Qualitative interviews | IFN | Psychological factors impacting on QOL for those with chronic HCV | Social, Physical |
| Davis and Rhodes (2004) | International Journal of Drug Policy | UK | Qualitative interviews | IFN | HCV risk perception and management | Cultural |
| Dowsett et al. (2017) | Canadian Journal of Gastroenterology & Hepatology |  | Systematic review and narrative synthesis | n/a | Lived experience of HCV | Physical, Cultural, Social |
| Dunne and Quayle (2001) | Journal of Health Psychology | Ireland | Qualitative Interpretative phenomenological analysis | IFN | Wellbeing and identity as a result of HCV | Physical |
| Falade-Nwulia et al. (2020) | International Journal of Drug Policy | US | Quantitative Cohort Survey | DAA | Impact of PWID social network members on HCV treatment | Social |
| Fraser and Treloar (2006) | Critical Public Health | Australia | Qualitative interview | IFN | Stigma and despair related to HCV diagnosis | Cultural |
| Giraudon et al. (2016) | Publication Office of the European Union |  | Report | DAA | Epidemiology, treatment and prevention of HCV | Cultural |
| Goodyear et al. (2020) | International Journal of Drug Policy | Canada | Qualitative interviews | DAA | Competing priorities, negative clinical encounters, complex health systems, stigma and opportunities for holistic approach | Cultural, Social |
| Goodyear et al. (2021a) | International Journal for Equity in Health | Canada | Qualitative interviews | DAA | Competing priorities, gate keeping and lack of information. Negative clinical encounters, scepticism, apprehension of side effects | Cultural, Social |
| Goodyear et al. (2021b) | International Journal of Drug Policy | Canada | Qualitative interviews | DAA | Improvement in physical health, hope and stigma. Employment and relationships, harm reduction | Social, Human and Physical |
| Grebely et al. (2019) | International Journal of Drug Policy |  | Editorial | n/a | Research priorities | Social |
| Groessl et al. (2008) | Journal of General Internal Medicine | USA | Qualitative interviews | IFN | Lived experience of HCV infection | Physical |
| Harris (2009a) | Body & Society | New Zealand | Qualitative interviews | n/a | Societal attitudes and stigma | Cultural, Social |
| Harris (2009b) | Sociology of health & illness | New Zealand and Australia | Qualitative interviews | IFN | Social acceptance of HCV and normalisation | Cultural, Social |
| Harris (2017) | International Journal of Drug Policy | UK | Qualitative longitudinal study | IFN & DAA | Treatment benefit from a patient perspective | Social, Human |
| Harris and Rhodes (2018) | International Journal of Drug Policy | UK | Qualitative ethnography | IFN & DAA | Non-clinical outcomes and transformation | Social |
| Madden et al. (2018) | Harm Reduction Journal | Australia | Qualitative interviews | DAA | Patient reported outcomes | Human |
| McDonald et al. (2013) | Journal of hepatology | UK | Quantitative Cross-sectional study | n/a | Quality of life related to HCV awareness | Cultural |
| Pourmarzi et al. (2020) | Australian Journal of Primary Health | Australia | Mixed-methods case study | DAA | Patient perceived outcomes of HCV treatment | Social |
| Rance et al. (2014) | Addiction | Australia | Qualitative evaluation | IFN | Identity, OST and HCV treatment | Social |
| Rance et al. (2021) | Health | Australia | Qualitative interviews | DAA | Pharmaceutical Citizenship, Politics of DAAs and complex long standing issues and stigma | Cultural |
| Razavi et al. (2020) | Liver International | 45 countries | Markov model forecasting  Report | DAA | Assessment of progress towards 2030 target | Cultural |
| Rhodes et al. (2013) | Sociology of Health & Illness | UK | Qualitative interviews | IFN | Patient citizenship | Cultural |
| Rhodes and Treloar (2008) | Addiction |  | Qualitative synthesis review | IFN | Social accommodation of HCV | Cultural |
| Richmond et al. (2018) | Hepatology, medicine & policy | Australia | Qualitative interviews | DAA | Patient experience of treatment | Social, Human |
| Skeer et al. (2018) | Drug and Alcohol Dependence | USA | Qualitative interviews | DAA | HCV knowledge and treatment uptake | Cultural |
| Torrens et al. (2020) | BMC infectious diseases | Europe | Quantitative Survey | DAA | Non-clinical outcomes of HCV treatment with DAAs | Human |
| Treloar et al. (2013) | Clinical infectious diseases |  | Review Article | IFN | Stigma as a barrier to HCV care and treatment | Cultural |
| Treloar and Rhodes (2009) | Qualitative Health Research |  | Qualitative synthesis review | IFN | Social stigma, biographical adaptation and medical and treatment encounters | Cultural, Social, Physical |
| Whiteley et al. (2016) | International Journal of Drug Policy | UK | Qualitative interviews | IFN and DAA | HCV treatment experience of interferon-free therapies | Cultural, Human |
| Whiteley et al. (2018) | Journal of Clinical Nursing | UK | Qualitative interviews | DAA | Experience of living with HCV in the DAA era. | Cultural, Human |
| Williams et al. (2019) | International Journal of Drug Policy | USA | Qualitative interviews | DAA | Social incentives of HCV treatment | Social, Human |
